# Cost–effectiveness of community-based practitioner programmes in Ethiopia, Indonesia and Kenya

**DOI:** 10.2471/BLT.14.144899

**Published:** 2015-08-03

**Authors:** Barbara McPake, Ijeoma Edoka, Sophie Witter, Karina Kielmann, Miriam Taegtmeyer, Marjolein Dieleman, Kelsey Vaughan, Elvis Gama, Maryse Kok, Daniel Datiko, Lillian Otiso, Rukhsana Ahmed, Neil Squires, Chutima Suraratdecha, Giorgio Cometto

**Affiliations:** a Nossal Institute for Global Health, University of Melbourne, Melbourne, Australia.; b Institute for International Health & Development, Queen Margaret University, Queen Margaret Drive Musselburgh, Edinburgh EH21 6UU, Scotland.; c Department of International Public Health, Liverpool School of Tropical Medicine, Liverpool, England.; d Royal Tropical Institute, Amsterdam, Netherlands.; e REACHOUT, Hidase Hulentenawi Agelglot Yebego Adragot Mahber, Awassa, Ethiopia.; f REACHOUT, LVCT Health, Nairobi, Kenya.; g Public Health England, North of England Region, England.; h United States Agency for International Development, Washington, DC, United States of America.; i Global Health Workforce Alliance, World Health Organization, Geneva, Switzerland.

## Abstract

**Objective:**

To assess the cost–effectiveness of community-based practitioner programmes in Ethiopia, Indonesia and Kenya.

**Methods:**

Incremental cost–effectiveness ratios for the three programmes were estimated from a government perspective. Cost data were collected for 2012. Life years gained were estimated based on coverage of reproductive, maternal, neonatal and child health services. For Ethiopia and Kenya, estimates of coverage before and after the implementation of the programme were obtained from empirical studies. For Indonesia, coverage of health service interventions was estimated from routine data. We used the Lives Saved Tool to estimate the number of lives saved from changes in reproductive, maternal, neonatal and child health-service coverage. Gross domestic product per capita was used as the reference willingness-to-pay threshold value.

**Findings:**

The estimated incremental cost per life year gained was 82 international dollars ($)in Kenya, $999 in Ethiopia and $3396 in Indonesia. The results were most sensitive to uncertainty in the estimates of life-years gained. Based on the results of probabilistic sensitivity analysis, there was greater than 80% certainty that each programme was cost-effective.

**Conclusion:**

Community-based approaches are likely to be cost-effective for delivery of some essential health interventions where community-based practitioners operate within an integrated team supported by the health system. Community-based practitioners may be most appropriate in rural poor communities that have limited access to more qualified health professionals. Further research is required to understand which programmatic design features are critical to effectiveness.

## Résumé

**Objectif:**

Évaluer le rapport coût-efficacité des programmes en faveur des praticiens communautaires en Éthiopie, en Indonésie et au Kenya.

**Méthodes:**

Le rapport coût-efficacité différentiel, pour les trois programmes, a été estimé selon une perspective gouvernementale. Des données sur les coûts ont été recueillies concernant l'année 2012. Les années de vie gagnées ont été estimées d'après l'offre de services dans le domaine de la santé génésique, maternelle, néonatale et infantile. Pour l'Éthiopie et le Kenya, les estimations de l'offre de services avant et après la mise en œuvre du programme ont été effectuées à partir d'études empiriques. Pour l'Indonésie, l'offre de services de soins a été estimée d'après des données de routine. Nous avons utilisé l'outil Lives-Saved Tool pour estimer le nombre de vies sauvées grâce aux changements intervenus dans l'offre de services en matière de santé génésique, maternelle, néonatale et infantile. Le produit intérieur brut par habitant a été pris comme seuil de référence de la disposition à payer.

**Résultats:**

Le coût différentiel estimé par année de vie gagnée était de 82 dollars internationaux ($) au Kenya, de 999$ en Éthiopie et de 3396$ en Indonésie. Les résultats étaient surtout sensibles à l'incertitude au niveau des estimations d'années de vie gagnées. D'après les résultats de l'analyse de sensibilité probabiliste, il était certain à plus de 80% que chaque programme présentait un bon rapport coût-efficacité.

**Conclusion:**

Les approches communautaires présentent vraisemblablement un bon rapport coût-efficacité pour la prestation de certains services de santé essentiels pour lesquels les praticiens communautaires interviennent dans le cadre d'une équipe intégrée appuyée par le système de santé. Les praticiens communautaires semblent être les plus indiqués dans les communautés rurales pauvres, qui ont un accès limité aux services de professionnels de santé plus qualifiés. Des recherches supplémentaires sont nécessaires pour déterminer les caractéristiques programmatiques qui sont cruciales pour l'efficacité des programmes.

## Resumen

**Objetivo:**

Evaluar la costoeficacia de los programas de médicos de ámbito comunitario en Etiopía, Indonesia y Kenya.

**Métodos:**

Se estimaron los porcentajes incrementales de costoeficacia para los tres programas desde un punto de vista gubernamental. Se recopilaron los datos de coste de 2012. Se estimaron los años de vida ganados en base a la cobertura de los servicios de salud reproductiva, materna, neonatal e infantil. En el caso de Etiopía y Kenya, las tasas de cobertura de antes y después de la implantación del programa se obtuvieron a través de estudios empíricos. En el caso de Indonesia, la cobertura de las intervenciones de los servicios de salud se estimó a través de datos rutinarios. Se utilizó la herramienta “Live Saved Tool” para estimar el número de vidas salvadas gracias al cambio en la cobertura de los servicios de salud reproductiva, materna, neonatal e infantil. El producto interior bruto per cápita se utilizó como el valor de umbral de referencia para la disposición a pagar.

**Resultados:**

El coste incremental estimado por año de vida ganado fue de 82 dólares internacionales ($) en Kenya, $999 dólares internacionales en Etiopía y $3.396 en Indonesia. Los resultados fueron más sensibles a la incertidumbre en las estimaciones de años de vida ganados. Basándose en los resultados de análisis de sensibilidad probabilísticos, hubo una certeza de más del 80% de que todos los programas eran costoeficaces.

**Conclusión:**

Es probable que los enfoques de ámbito comunitario sean costoeficaces para suministrar algunas intervenciones sanitarias esenciales en los lugares en los que los médicos de ámbito comunitario operan dentro de un grupo integrado apoyado por el sistema sanitario. Los médicos de ámbito comunitario pueden ser más apropiados en comunidades rurales pobres que tengan acceso limitado a profesionales de la salud más cualificados. Se requiere de más investigación para comprender qué características de diseño programático son cruciales para la efectividad.

## ملخص

الغرض تقييم الفعالية من حيث التكلفة لبرامج العاملين في الخدمات الصحية المجتمعية في إثيوبيا وإندونيسيا وكينيا.

الطريقة تم تقييم معدلات تزايدية للفعالية من حيث التكلفة للبرامج الثلاثة من منظور حكومي. وتم تجميع بيانات التكلفة لعام 2012. كما تم تقدير سنوات العمر المكتسبة بناءً على تغطية خدمات الصحة الإنجابية وصحة الأمهات والحوامل والأطفال حديثي الولادة والأطفال صغار السن. بالنسبة إلى إثيوبيا وكينيا، فقد تم استخلاص التقديرات الخاصة بالتغطية قبل تنفيذ البرنامج وبعده من واقع الدراسات التجريبية. أما بالنسبة إلى إندونيسيا، فقد تم وضع التقدير الخاص بتغطية برامج التدخل للخدمات الصحية من واقع البيانات الروتينية. وقد اعتمدنا على استخدام الأداة الخاصة بحساب عدد الأرواح التي تم إنقاذها لتقدير عدد أرواح البشر الذين تم إنقاذهم من خلال التغييرات التي طرأت على التغطية الصحية لخدمات الصحة الإنجابية وصحة الأمهات والحوامل والأطفال حديثي الولادة والأطفال صغار السن. وتم الاعتماد على نصيب الفرد من إجمالي الناتج المحلي كقيمة حدية مرجعية للاستعداد للدفع. ****

النتائج بلغت التكلفة التزايدية التقديرية لكل سنة عمر تم اكتسابها 82 دولارًا دوليًا في كينيا، و999 دولار دولي في إثيوبيا، و3396 دولارًا أمريكيًا في إندونيسيا. وكانت النتائج أكثر تأثرًا بعدم اليقين السائد في التقديرات الخاصة بسنوات العمر المكتسبة. وبناءً على نتائج تحليل احتمال التأثر، فقد زادت نسبة اليقين عن 80% من أن كل برنامج كان فعالاً من حيث التكلفة. ****

الاستنتاج من المرجح أن تكون الأساليب المنهجية المجتمعية فعالة من حيث التكلفة لتقديم بعض برامج التدخل الصحي الضرورية والتي يباشر فيها العاملون في مجال الخدمات الصحية أعمالهم في إطار فريق متكامل يستند إلى الدعم المقدم من النظام الصحي. قد يكون العاملون في مجال الخدمات الصحية هم الأنسب في المجتمعات الريفية الفقيرة والتي تتوفر فرص محدودة للاستفادة من خدمات أخصائيين صحيين مؤهلين. ولذلك، يلزم إجراء المزيد من الأبحاث لاستيعاب خصائص تصميم البرامج الضرورية لتحقيق الكفاءة. ****

## 摘要

**目的:**

评估埃塞俄比亚、肯尼亚和印度尼西亚社区医生项目的成本效益。

**方法:**

从政府角度估计三个项目的增量成本效益。 收集 2012 年的成本数据。根据生育、孕产妇、新生儿和儿童保健服务的覆盖范围估计挽救的生命年。 通过实证研究估计埃塞俄比亚和肯尼亚在项目实施前和实施后的覆盖范围。 通过常规数据估计印度尼西亚卫生服务干预的覆盖范围。 我们使用挽救的生命计算工具估计由于生育、孕产妇、新生儿和儿童保健服务覆盖范围的变化挽救的生命数量, 使用人均国内生产总值作为参考支付意愿阈值。

**结果:**

埃塞俄比亚、肯尼亚和印度尼西亚挽救的生命年每一年估计增量成本分别是 999 国际元、82 国际元和 3396 国际元。 这些结果对于挽救的生命年估计的不确定性最为敏感。 根据概率敏感度分析结果,每一个具有成本效益的项目中均存在 80% 以上的确定性。

**结论:**

基于社区的方法可能会提高推行一些重要的卫生干预措施的成本效益,通过卫生干预措施,社区医生可以在卫生体系支持的综合服务团队中行医。 社区医生是缺乏合格的卫生专业人员的农村贫困群体最合适的就医选择。 理解哪些项目设计特点对于成本效益至关重要,仍需进一步研究。

## Резюме

**Цель:**

Оценить рентабельность программ общинной медицинской помощи в Эфиопии, Индонезии и Кении

**Методы:**

Коэффициенты эффективности дополнительных расходов были оценены для трех программ с точки зрения правительства. Данные по расходам собирались в течение 2012 года. Прирост продолжительности жизни оценивался на основании охвата населения услугами в области охраны репродуктивного здоровья, здоровья матерей, новорожденных и детей. Для Эфиопии и Кении оценка охвата до и после внедрения программы была получена в ходе эмпирических исследований. Для Индонезии охват населения соответствующими услугами здравоохранения оценивался по регулярно поступающим данным. Для оценки количества жизней, сохраненных в результате расширения охвата населения услугами в области охраны репродуктивного здоровья, здоровья матерей, новорожденных и детей, использовалось средство вычисления прироста жизни. В качестве порогового значения готовности оплачивать услуги рассматривался валовой внутренний продукт на душу населения.

**Результаты:**

По оценкам прирост расходов при увеличении срока жизни на год составил 82 международных доллара в Кении, 999 международных долларов в Эфиопии и 3 396 международных долларов в Индонезии. Результаты были больше всего чувствительны к неопределенности в оценке количества дополнительных лет жизни. На основании вероятностного анализа чувствительности можно более чем с 80%-ной уверенностью утверждать, что каждая программа была рентабельной.

**Вывод:**

При оказании некоторых наиболее необходимых услуг медицинской помощи ориентированный на общины подход вероятнее всего будет рентабелен в том случае, когда живущие в той или иной общине врачи составляют единую команду, поддерживаемую системой здравоохранения. Такая практика больше подходит для бедных сельских общин, в которых доступ к более квалифицированной медицинской помощи затруднен. Необходимы дополнительные исследования для понимания того, какие именно характеристики программ оказываются критическими для достижения ими рентабельности.

## Introduction

Community-based strategies have the potential to expand access to essential health services, especially in light of critical shortages in the health workforce.^1^ The term community health worker has been used to refer to volunteers and salaried, professional or lay health workers with a wide range of training, experience, scope of practice and integration in health systems. In the context of this study, we use the term community-based practitioner to reflect the diverse nature of this group of health workers.

Community-based practitioners have been found to be effective in delivering health services in low- and middle-income countries.^2^^–^^6^ A common premise is that community-based practitioners are more responsive to the health needs of local populations than clinic-based services, are generally less expensive and can promote local participation in health. They can also improve coverage and health equity for populations that are difficult to reach with clinic-based approaches.^7^^–^^9^

The aim of the present study is to assess the cost–effectiveness of community-based practitioner programmes with different design features across three countries – Ethiopia, Indonesia and Kenya – in which these initiatives have been implemented to scale.

### Programme description

Globally, many different types of community-based practitioner programmes have evolved since 1978, when the first international conference on primary health care was held in Alma Ata, Kazakhstan, in the former Soviet Union. Community-based practitioners may operate in the public or private sectors and respond to single or multiple health issues.^10^^,^^11^ Specific design features of community-based programmes that work in one context may not work in another. The programmes described here differ markedly in their design, including the type of worker, level of training, scope of work, nature of supervision and the extent to which basic equipment is provided (Table 1).

**Table 1:** Community-based practitioners programmes in Ethiopia, Indonesia and Kenya

Feature	Ethiopia	Indonesia	Kenya
Start, year	2004	1989	2006
Focus area	Maternal and child health (including antenatal, safe and clean delivery at the health post, immunization, growth monitoring and nutritional advice), family planning, immunization, adolescent reproductive health and nutrition	Maternal health: antenatal care, point-of-care tests e.g. malaria (in endemic regions) and HIV (only in Papua region), treatment such as for malaria, outreach care and providing safe delivery within a health facility and at home, postnatal checks, immunization	Maternal and child health prevention and promotion activities that link community members to the health system (registration, education, referral, follow-up)
Name of community-based practitioner	Health extension worker	Village midwives	Community health workers
Corresponding category in ILO’s ISCO	3253 (community health workers)	3222 (midwifery associate professional)	3253 (community health workers)
Type of volunteers	Voluntary community health promoters	Community health volunteers and traditional birth attendants	None
Population catchment area	2 workers for 5000 people	1 worker per village of 500–1500 people	50 workers for 5000 people
Primary base of service delivery	A local health post but spend 70% of their time on house-to-house visits	Sub-health posts and village clinics	Community (home visits)
Initial training	1 year (government funded)	Nursing academy 3 years (self-funded)	10 days training (government funded)
One-off incentive kits	Backpacks	Motorbikes	Backpacks
Salary	Annual salary of approximately $2400	Annual salary of approximately $4250	Unpaid
Other financial incentives and allowances	None	Transport allowances; incentive per antenatal care, delivery assisted and postnatal care	None
In-service training	On-job training in relation to local interventions	Refresher training offered (but none administered in the district in 2012)	Quarterly updates (but none administered in the district in 2012)
Supervision structure	Supervised by health centre and district health office personnel	Supervised by health centre and district health office personnel	Supervised by health centre personnel – community health extension workers at health centre level

HIV: human immunodeficiency virus; ILO: International Labour Organization; ISCO: International Standard Classification of Occupations.

Note: Categories of programme have been developed by the REACHOUT consortium http://www.reachoutconsortium.org.

Ethiopia launched its health extension programme in 2004 with a view to achieving universal coverage of primary health care.^12^ Districts with five to seven health centres are divided into administrative units covering a population of 5000 people, each with a health post staffed by two health extension workers. Health extension workers are women, trained and salaried by the government, who work in the community delivering primary health services and are trained to administer basic medicines and vaccines.

In Indonesia, the health system is decentralized with an emphasis on community health care.^13^ Primary maternal and child health-care services are provided at community health centres with services extended through village health posts, village birthing facilities and monthly outreach events. In each village, a trained midwife or nurse is assisted by community health volunteers who provide primary health care with a focus on prevention and health promotion activities.^14^

In Kenya, there are four tiers of service provision – community, primary care, primary (county) referral and tertiary (national) referral services.^15^ The Kenya community health strategy, rolled out in 2006,^16^ stipulates that community health services should provide services to community units of 5000 people, with each unit covered by 50 volunteer community-based practitioners, each responsible for disease prevention and control in 20 households. These community-based practitioners are linked to primary health facilities and supervised by government-employed community health extension workers.

## Methods

We estimated incremental cost–effectiveness ratios for community-based practitioner programmes, using data from four districts: Shebedino (Ethiopia), south-west Sumba (Indonesia), Takala (Indonesia) and Kasarani (Kenya). In Indonesia, two districts were chosen to better reflect the diversity of context and programme implementation in that country. The main inclusion criteria for country selection were that programmes should be national in scale, performing similar activities and with data available on effectiveness.

We assessed the cost–effectiveness of each programme from a government perspective. Costs and lives saved were estimated over a one-year time period. We assumed that all costs and benefits were additional to those that would have occurred in the absence of the new programme (Table 2).

**Table 2:** Model assumptions

	Model assumptions
Time horizon	A one year time horizon was assumed
Discount rate	3% discount rate was applied for start-up costs and life years gained
Useful life of programme	10 years was applied in estimating annual equivalent costs
Attrition rate	Attrition rate was assumed to be 0% for Kenya and Indonesia
Overhead cost	An overhead cost of 15% was assumed
One way sensitivity analysis	The one-way sensitivity analysis was performed by varying all model inputs by ± 30%
Probabilistic sensitivity analysis	Model inputs were varied by ± 10%. Gamma distributions were specified for all cost inputs. Beta distributions were specified for attrition rate and overhead cost percentage. Normal distribution was specified for life years gained

### Measurement of effectiveness

Disability-adjusted life years and quality-adjusted life years have been widely used as measures of the effectiveness of health programmes. However, the disability and utility weights required to quantify these outcomes were not available for our study outcomes. We used life-years gained (LYG) as our measure of effectiveness. LYG is a validated measure of population health;^17^ though it does not account for quality of life, it is suitable for this study given the data available.

We used the Lives Saved Tool (LiST)^18^ to estimate the number of lives saved due to changes in coverage of reproductive, maternal, neonatal and child health interventions. The Lives Saved Tool models the impact of scaling-up the coverage of proven interventions on maternal, neonatal and child mortality by integrating evidence on intervention effectiveness^19^^,^^20^and demographic projections of mortality.

To estimate the number of lives saved, we adjusted coverage data to a target level of coverage. For Ethiopia and Kenya, target coverage data were obtained from empirical studies evaluating the impact of each country’s programme.^21^^–^^23^ For Indonesia, coverage data were obtained from routine data reported by village midwives.

The Lives Saved Tool uses national demographic data to produce estimates of lives saved in a national population. Therefore, national estimates of lives saved were scaled down to district level based on the proportion of the national population in each study district. We classified lives saved in four age groups: live births; children younger than 1 month; children aged between 1 and 59 months and mothers. For each category, the number of lives saved was multiplied by the remaining life expectancy at the time death was averted. The resulting LYG were discounted using a 3% annual discount rate.^24^ Remaining life expectancies were obtained from life tables.^25^

### Cost estimates

The financial cost (for the year 2012 or earlier where necessary) of each programme was estimated from data collected between August and September 2013 from each country. Local currencies were converted to international dollars using purchasing power parity exchange rates (available at http://data.worldbank.org/indicator/PA.NUS.PPP). We report all cost data in international dollars ($). Cost data included start-up costs and recurrent costs. Equivalent annual costs were estimated by annuitizing total start-up cost based on a useful life of 10 years and a 3% discount rate.^24^ In the Ethiopian model, an attrition rate of 1.1% was applied to account for attrition after training of community-based practitioners. However, due to lack of relevant data, the attrition rate was assumed to be zero in the Indonesian and Kenyan models. Recurrent costs were estimated based on operational processes of the programme in 2012 and combined with annual start-up costs to obtain estimates of total annual cost of the programme. Overhead costs equivalent to 15% were added to account for cost incurred at higher administrative levels.^26^ Incremental cost of medicines and vaccines attributed to changes in coverage of reproductive, maternal, neonatal and child interventions were included for only the Ethiopian model but excluded from the Kenyan and Indonesian models due to lack of data. Unit cost data were collected from a variety of sources including expenses files, health workers’ payroll records, key informant interviews and supply catalogues for medicines and supplies.^27^

For all districts, incremental cost–effectiveness ratios were expressed as incremental cost per LYG; the detailed cost–effectiveness model is available from the authors. Cost–effectiveness was assessed using each country’s national gross domestic product (GDP) per capita as the reference willingness-to-pay threshold value.^28^

### Sensitivity analyses

We did two sensitivity analyses. First, we did a univariate sensitivity analysis. The impact of each model parameter (costs, LYG, attrition rate, discount rate, percent overhead cost and useful life of programme), on the results was assessed by sequentially varying each parameter over a specified range (± 30%) while holding the other parameters constant. Second, we did a probabilistic sensitivity analysis. An appropriate probability distribution was fitted around each parameter mean and varied within lower and upper bounds (± 10). All cost inputs were specified as gamma distributions; LYG was specified as a normal distribution and attrition rate and percentages (used in estimating overhead costs) were specified as beta distributions.^29^ Parameter uncertainty was propagated through the model using 5000 Monte Carlo simulations and the results presented as cost–effectiveness acceptability curves.

## Results

### Programme effects

Coverage and change in coverage of interventions affected by the programme are shown in Table 3. We used these results to calculate the number of lives saved. Overall, the numbers of lives saved increased in all districts, varying from 5.78 lives saved per 100 000 population in south-west Sumba to 26.33 lives saved per 100 000 population in Kasarani. In Shebedino, more children’s lives were saved in the older cohort (1–59 months) compared to the younger cohort (younger than 1 month). Conversely, in south-west Sumba, Takala and Kasarani districts, more lives were saved in the younger cohort, compared to the older cohort (Table 4).

**Table 3:** Interventions and effectiveness of community-based practitioners programmes, Ethiopia, Indonesia and Kenya, 2007–2012

Intervention	Shebedino, Ethiopia (2007 & 2010)	Sumba, Indonesia (2012)	Takala, Indonesia (2012)	Kasarani, Kenya (2010)	
	Coverage change (%)	Coverage (%)	Coverage (%)	Coverage change (%)	
Pregnancy					
Antenatal care	8.9	45.2	96. 0	23. 0	
Tetanus toxoid administration	7.0	–	96. 0	–	
Iron folate supplementation	7.4	88.6	98. 0	–	
Childbirth					
Skilled birth attendance	–	50.5	92. 0	26. 0	
Breastfeeding					
Promotion of breastfeeding	8.4	–	–	32. 0	
Postnatal care					
Preventive postnatal care	11.2	65.9	100. 0	–	
Others					
Hygienic disposal of children’s faeces	1.1	–	–	–	
Household ownership of ITN	7.9	–	–	–	
Vaccines				–	
BCG	9.3	–	–	–	
Polio	9.1	–	–	–	
DPT	11.6	–	–	–	
Measles	11.8	–	–	–	
	**Lives saved**			
**National population**	5 299	13 930	58 471	11 894	
**Study population**	17	16	65	1.3	

BCG: bacille Calmette-Guérin; DPT: diphtheria-pertussis-tetanus; ITN: insecticide-treated bed net.

Sources: Ethiopia^21^^,^^22^; Indonesia: routine data reported by village midwives ; Kenya.^23^

**Table 4:** Effectiveness of community-based practitioners programmes by district and population group in Ethiopia, Indonesia and Kenya, 2012

District, country	Population group	Lives saved		Life years gained^b^
		Total	per 100 000 population^a^	
Shebedino, Ethiopia	Still birth	5.40	1.94	151
	< 1 month	4.21	1.52	117
	1–59 months	7.18	2.58	203
	Maternal	0.01	0.005	0
	**Total**	**16.80**	**6.05**	**471**
Sumba, Indonesia	Still birth	2.22	0.78	65
	< 1 month	12.76	4.50	373
	1–59 months	−0.04	−0.01	−1
	Maternal	1.44	0.51	38
	**Total**	**16.38**	**5.78**	**475**
Takala, Indonesia	Still birth	24.73	9.17	722
	< 1 month	35.55	13.19	1038
	1–59 months	−0.24	−0.09	−7
	Maternal	5.31	1.97	142
	**Total**	**65.35**	**24.24**	**1894**
Kasarani, Kenya	Still birth	0.41	8.22	11
	< 1 month	0.74	14.88	21
	1–59 months	0.05	0.96	1
	Maternal	0.11	2.27	3
	**Total**	**1.31**	**26.33**	**36**

^a^ There were 277 788 people in Shebedino, 283 818 people in south-west Sumba, 269 603 people in Takala and 5000 people in Kasarani.

^b^ Totals may differ due to rounding

### Costs

Costs differed across the countries, reflecting differences in the design and operational features of the programmes (Table 5, available at: http://www.who.int/bulletin/volumes/93/9/14-144899). For example, pre-service training costs were considerably higher in Ethiopia compared to Kenya, capturing differences in the length of pre-service training (1 year in Ethiopia versus 10 days in Kenya). Annual salary costs for Indonesia were considerably higher than in Ethiopia, reflecting differences in the educational attainment between the community-based practitioners and local economic factors. In Kenya, cost of stationery and registers contributes the highest proportion to total cost accounting for over 50% of total cost. This reflects the low level of other costs including the volunteer status of the practitioners in Kenya and the government perspective taken.

**Table 5:** Costs of community-based practitioners programmes, in international dollars, Ethiopia, Indonesia and Kenya, 2012

Cost category	Shebedino, Ethiopia	Sumba, Indonesia	Takala, Indonesia	Kasarani, Kenya
**Start-up cost^a^**				
Pre-service training	8 848	–	5 383	729
One-off incentives/starter kits	84	7 390	11 381	233
Construction of new health posts	83 806	817 593	668 940	–
Equipment	15 437	5 213	12 284	25
Total start-up costs	108 515	830 196	697 988	988
**Direct recurrent cost**				
Annual salary of community-based practitioners	181 094	323 471	762 248	–
In-service training	16 303	35 620	1 484	–
Other monetary incentives and allowances	–	254 398	2 334 921	–
Medicines^b^	13 413	–	–	–
Stationery (registers, books)	–	38 579	38 579	1 552
Total direct recurrent costs	210 810	652 069	3 137 232	1 552
**Indirect recurrent costs**				
Supervisory visits	97 409	5 964	3 460	186
Supervisory meetings	7 245	259	10 715	–
Total indirect recurrent costs	104 654	6 223	14 174	186
**Other costs**				
Total volunteer costs	–	21 646	310 521	–
Overhead costs	47 320	101 991	519 289	261
**Total cost**	**470 958**	**1 612 125**	**4 679 205**	**2 986**

^a^ Total cost annuitized based on 10 years useful life of programme and 3% discount rate.

^b^ Only cost of medicines and vaccines for which available estimates of changes in coverage are attributable to the community-based practitioners programme were included. These data were only available for the Ethiopian model.

Notes: Cost is estimated on the basis of 75 community-based practitioners in Shebedino; 76 community-based practitioners and 2315 volunteers and traditional birth attendants in south-west Sumba; 182 community-based practitioners and 2298 volunteers and traditional birth attendants in Takala; and 50 community-based practitioners in Kasarani. Totals may differ due to rounding.

### Cost–effectiveness

Incremental costs per LYG were $999 in Shebedino, $3396 in south-west Sumba, $2470 in Takala and $82 in Kasarani (Table 6). All three programmes were cost-effective when using the willingness-to-pay threshold value as a reference.

**Table 6:** Cost–effectiveness of community-based practitioners programmes, Ethiopia, Indonesia and Kenya, 2012

	Shebedino, Ethiopia	Sumba, Indonesia	Takala, Indonesia	Kasarani, Kenya
Incremental cost, $	470 958	1 612 125	4 679 205	2 986
Life years gained	471	475	1 894	36
ICER (range), $/LYG	999 (998–1 001)	3 396 (3 391–3 402)	2 470 (2 469−2 477)	82 (82–82)

ICER: incremental cost–effectiveness ratio; LYG: life years gained; $: international dollars.

**Fig. 1:**
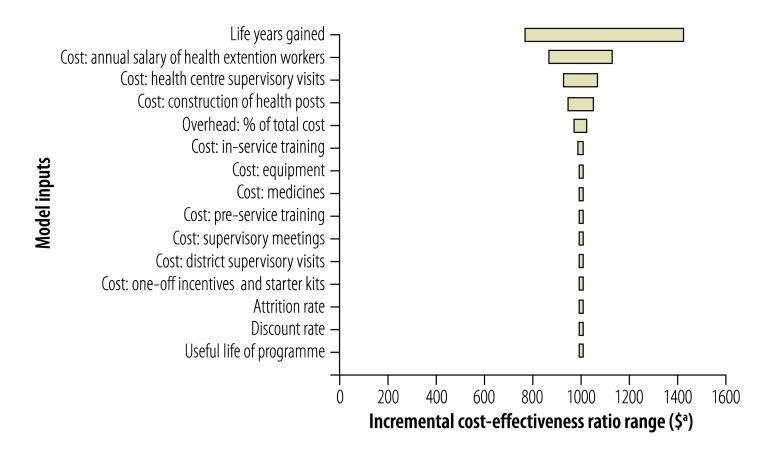
Sensitivity analysis, Shebedino district, Ethiopia

**Fig. 2:**
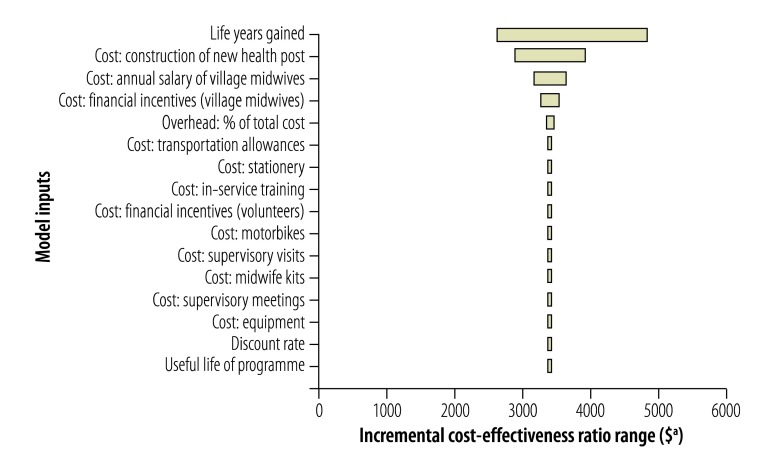
Sensitivity analysis, Sumba district, Indonesia

**Fig. 3:**
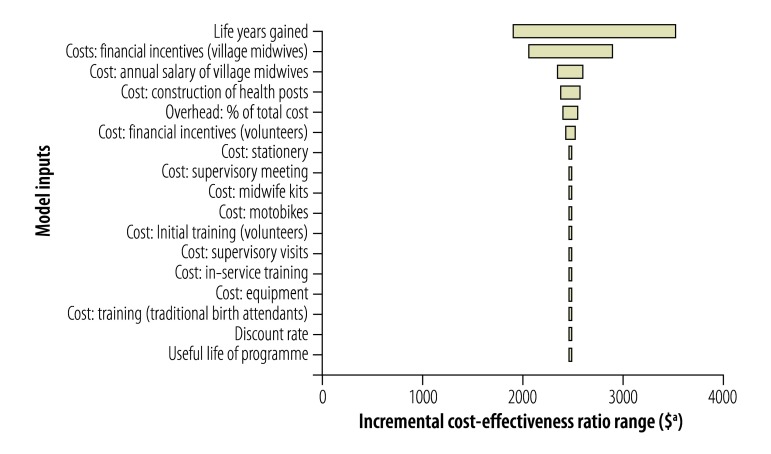
Sensitivity analysis, Takala district, Indonesia

**Fig. 4:**
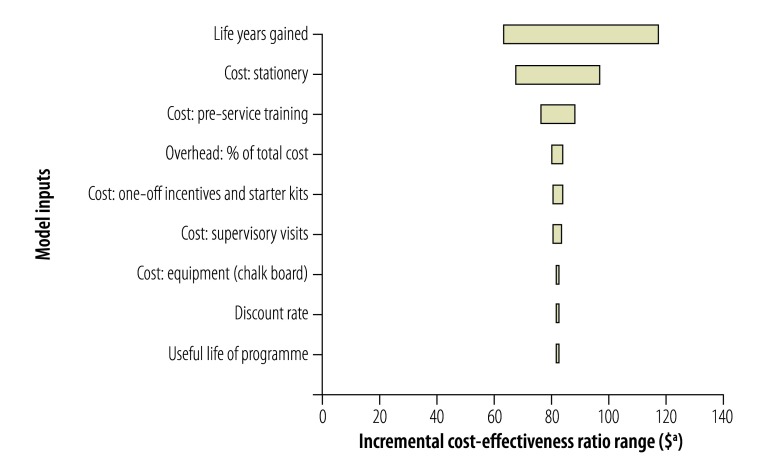
Sensitivity analysis, Kasarani district, Kenya

Univariate sensitivity analyses (Fig. 1, Fig. 2, Fig. 3, Fig. 4) show that cost–effectiveness is most sensitive to uncertainties in the estimates of LYG. The probabilistic sensitivity analyses suggested that the programmes in all four study districts are likely to be cost-effective (> 80% probability) assuming a willingness-to-pay threshold of one to three times each country’s GDP per capita.

**^a^**International dollars, 2012.

**^a^**International dollars, 2012.

**^a^**International dollars, 2012.

**^a^**International dollars, 2012.

## Discussion

Given the assumptions made, we find each community-based practitioner programme to be cost-effective and to improve coverage of essential services. Several studies have also found a variety of community-based programmes to be cost-effective compared to facility-based interventions delivered by other types of health workers.^5^^, ^^30^^–^^32^ Cost–effectiveness was most sensitive to uncertainty in the estimation of LYG. Given that LYG were estimated indirectly from coverage data or in the case of Kenya from potentially less robust evidence on coverage change, further research on the effectiveness of community-based practitioner programmes should be a priority.

The community-based practitioner programmes in the four study districts appear to have contributed to saving lives. However, there were differences across population categories which can be explained by differences in the reproductive, maternal, neonatal, and child health interventions used to estimate the additional lives saved. In south-west Sumba, Takala, and Kasarani districts, data on the effect of the community-based practitioner programme were only available for interventions targeting neonatal health. In Shebedino district, data were available mostly for interventions targeting the health of older children.

The analysis has several limitations. It is possible that by choosing programmes for which some effectiveness evidence was available, well-functioning programmes may have been selected. On the other hand, the approach used may have underestimated cost–effectiveness, since it was not possible to capture the full range of effects produced by community-based practitioners. Although community-based practitioners address a wide range of health conditions in different contexts, this study restricted the assessment to interventions with clear health benefits. In theory, a broader assessment of the impact might have increased the effectiveness of the community-based practitioner programmes under study, by capturing their positive contribution in other health services areas, as well as other domains, including reduced morbidity and wider social benefits.

We may have under or overestimated cost–effectiveness by using a government rather than a societal approach; neither societal costs nor potential societal benefits were captured in this study. We did not account for possible interactions between the new community-based practitioner programmes and other established health system features. This has implications for estimates of the incremental costs and benefits of the community-based practitioner programmes assessed.

For Ethiopia and Kenya, there was a mismatch in the time periods from which cost and effectiveness data were obtained, since we relied on evidence of effectiveness from historical studies. Furthermore, a one year time horizon may bias incremental cost–effectiveness estimates for newly implemented programmes whose benefits are only fully realized several years after implementation.^33^ However, this is unlikely to be the case in this study given that the programmes analysed have been implemented at scale for years and are well established.

We cannot answer several policy-relevant questions concerning the design, use and scale-up of community-based practitioner initiatives. This is because there is limited empirical evidence on the influence of different design features (e.g. contents and duration of training, amount and type of supervision, or level of remuneration). Volunteer community-based practitioners describe a range of motivations, many of which are intrinsic and relate to personal, family or community value systems.^34^ However this does not preclude the desire for financial remuneration and for predictability of payments.^35^ Community health strategies that are highly dependent on volunteers tend to have high attrition rates, lower reporting and intermittent attendance at supervision.^36^ For example, in Kenya, if reliable data about these factors and their implications had been available and included, using volunteers may not have been as cost-effective as our model suggests. Reimbursement and volunteering raise complex ethical and economic questions,^37^ which have led to a revision in Kenya’s community health strategy.^38^

There is growing awareness that delegating tasks to community-based practitioners with shorter training is not a sufficient answer to the health workforce challenges faced by many health systems. Effective task sharing requires a comprehensive and integrated reconfiguration of health-care teams, a revision in their scope of practice and supportive regulatory frameworks.^9^ In contexts where community-based practitioners operate within an integrated team supported by the health system, community-based approaches are likely to be cost-effective for delivery of some essential health interventions. However, it should not be assumed that initiatives disjointed from health system support or with radically different design features than those described in this study are equally cost-effective. Overall, community-based practitioners should not be seen as a low-cost alternative to the provision of standard care, but rather a complementary approach of particular relevance in rural poor communities that have limited access to more qualified health professionals.

There is an opportunity to accelerate progress towards universal health coverage by integrating community-based practitioners in national health-care systems.^39^ However, more attention needs to be given to understanding costs and cost–effectiveness from both a government and societal perspective, especially in a policy context in which there are growing calls for scaling up these programmes.^1^ There are numerous policy issues that neither our study nor the available research can adequately address, such as how context and design elements affect cost–effectiveness. Mixed methods research is needed to develop a more nuanced understanding of the determinants of the costs and effectiveness of community-based practitioner programmes in different contexts.

## References

[1] Singh P, Sachs JD. 1 million community health workers in sub-Saharan Africa by 2015. Lancet. 2013 7 27;382(9889):363–5. 10.1016/S0140-6736(12)62002-9 23541538

[2] Gilmore B, McAuliffe E. Effectiveness of community health workers delivering preventive interventions for maternal and child health in low- and middle-income countries: a systematic review. BMC Public Health. 2013;13(1):847. 10.1186/1471-2458-13-847 24034792 PMC3848754

[3] Glenton C, Scheel IB, Lewin S, Swingler GH. Can lay health workers increase the uptake of childhood immunisation? Systematic review and typology. Trop Med Int Health. 2011 9;16(9):1044–53. 10.1111/j.1365-3156.2011.02813.x 21707877

[4] Lewin S, Munabi-Babigumira S, Glenton C, Daniels K, Bosch-Capblanch X, van Wyk BE, et al. Lay health workers in primary and community health care for maternal and child health and the management of infectious diseases. Cochrane Database Syst Rev. 2010; (3):CD004015. 20238326 10.1002/14651858.CD004015.pub3 PMC6485809

[5] Perry H, Zulliger R. How effective are community health workers? An overview of current evidence with recommendations for strengthening community health worker programs to accelerate progress in achieving the health-related Millennium Development Goals. Baltimore: Johns Hopkins Bloomberg School of Public Health; 2012.Available from: http://www.coregroup.org/storage/Program_Learning/Community_Health_Workers/review%20of%20chw%20effectiveness%20for%20mdgs-sept2012.pdf[cited 2015 Aug 13].

[6] van Ginneken N, Tharyan P, Lewin S, Rao GN, Meera SM, Pian J, et al. Non-specialist health worker interventions for the care of mental, neurological and substance-abuse disorders in low- and middle-income countries. Cochrane Database Syst Rev. 2013;11:CD009149. 24249541 10.1002/14651858.CD009149.pub2

[7] Carrera C, Azrack A, Begkoyian G, Pfaffmann J, Ribaira E, O’Connell T, et al.; UNICEF Equity in Child Survival, Health and Nutrition Analysis Team. The comparative cost-effectiveness of an equity-focused approach to child survival, health, and nutrition: a modelling approach. Lancet. 2012 10 13;380(9850):1341–51. 10.1016/S0140-6736(12)61378-6 22999434

[8] Chopra M, Sharkey A, Dalmiya N, Anthony D, Binkin N; UNICEF Equity in Child Survival, Health and Nutrition Analysis Team. Strategies to improve health coverage and narrow the equity gap in child survival, health, and nutrition. Lancet. 2012 10 13;380(9850):1331–40. 10.1016/S0140-6736(12)61423-8 22999430

[9] Lehmann U, Van Damme W, Barten F, Sanders D. Task shifting: the answer to the human resources crisis in Africa? Hum Resour Health. 2009;7(1):49. 10.1186/1478-4491-7-49 19545398 PMC2705665

[10] Bloom GHS. Pluralism and marketisation in the health sector: meeting health needs in contexts of social change in low and middle income countries. Brighton: Institute of Development Studies; 2001.

[11] Standing H, Chowdhury AMR. Producing effective knowledge agents in a pluralistic environment: what future for community health workers? Soc Sci Med. 2008 5;66(10):2096–107. 10.1016/j.socscimed.2008.01.046 18342421

[12] Teklehaimanot HD, Teklehaimanot A. Human resource development for a community-based health extension program: a case study from Ethiopia. Hum Resour Health. 2013;11(1):39. 10.1186/1478-4491-11-39 23961920 PMC3751859

[13] Heywood P, Choi Y. Health system performance at the district level in Indonesia after decentralization. BMC Int Health Hum Rights. 2010;10(1):3. 10.1186/1472-698X-10-3 20205724 PMC2839983

[14] Dawson A, Howes T, Gray NEK. Human resources for health in maternal, neonatal and reproductive health at community level: a profile of Indonesia. Sydney: Human Resources for Health Knowledge Hub, University of New South Wales; 2011.

[15] Kenya Health Policy 2012–2030. Nairobi: Ministry of Medical Services and Ministry of Public Health and Sanitation; 2012.

[16] Taking the Kenya Essential Package for Health to the Community: a strategy for the delivery of level one services. Nairobi: Ministry of Health; 2006.

[17] Robberstad B. QALYs vs DALYs vs LYs gained: What are the differences and what difference do they make for health care priority setting? Nor Epidemiol. 2005;15(2):183–91.

[18] Winfrey W, McKinnon R, Stover J. Methods used in the Lives Saved Tool (LiST). BMC Public Health. 2011;11 Suppl 3:S32. 10.1186/1471-2458-11-S3-S32 21501451 PMC3231906

[19] Jones G, Steketee RW, Black RE, Bhutta ZA, Morris SS; Bellagio Child Survival Study Group. How many child deaths can we prevent this year? Lancet. 2003 7 5;362(9377):65–71. 10.1016/S0140-6736(03)13811-1 12853204

[20] Walker N, Fischer-Walker C, Bryce J, Bahl R, Cousens S; CHERG Review Groups on Intervention Effects. Standards for CHERG reviews of intervention effects on child survival. Int J Epidemiol. 2010 4;39 Suppl 1:i21–31. 10.1093/ije/dyq036 20348122 PMC2845875

[21] Karim AM, Admassu K, Schellenberg J, Alemu H, Getachew N, Ameha A, et al. Effect of Ethiopia’s health extension program on maternal and newborn health care practices in 101 rural districts: a dose-response study. PLoS ONE. 2013;8(6):e65160. 10.1371/journal.pone.0065160 23750240 PMC3672192

[22] Admassie A, Abebaw D, Woldemichael AD. Impact evaluation of the Ethiopian Health Services Extension Programme. Journal of Development Effectiveness. 2009;1(4):430–49. 10.1080/19439340903375724

[23] Wangalwa G, Cudjoe B, Wamalwa D, Machira Y, Ofware P, Ndirangu M, et al. Effectiveness of Kenya’s Community Health Strategy in delivering community-based maternal and newborn health care in Busia County, Kenya: non-randomized pre-test post test study. Pan Afr Med J. 2012;13(12) Suppl 1:12. 23467438 PMC3587017

[24] Johns B, Baltussen R, Hutubessy R. Programme costs in the economic evaluation of health interventions. Cost Eff Resour Alloc. 2003 2 26;1(1):1. 10.1186/1478-7547-1-1 12773220 PMC156020

[25] Life tables by country [Internet]. Geneva: World Health Organization; 2015. Available from: http://apps.who.int/gho/data/node.main.692?lang=en [cited 2015 Jun 30].

[26] McCord GC, Liu A, Singh P. Deployment of community health workers across rural sub-Saharan Africa: financial considerations and operational assumptions. Bull World Health Organ. 2013 4 1;91(4):244–53B. 10.2471/BLT.12.109660 23599547 PMC3629450

[27] Supply catalogue. New York: United Nations Children’s Fund; 2012.

[28] Macroeconomics and health: investing in health for economic development. Report of the Commission on Macroeconomics and Health. Geneva: World Health Organisation; 2001.

[29] Briggs AH. Handling uncertainty in cost-effectiveness models. Pharmacoeconomics. 2000 5;17(5):479–500. 10.2165/00019053-200017050-00006 10977389

[30] Chanda P, Hamainza B, Moonga HB, Chalwe V, Banda P, Pagnoni F. Relative costs and effectiveness of treating uncomplicated malaria in two rural districts in Zambia: implications for nationwide scale-up of home-based management. Malar J. 2011;10(1):159. 10.1186/1475-2875-10-159 21651828 PMC3121654

[31] Datiko DG, Lindtjørn B. Cost and cost-effectiveness of treating smear-positive tuberculosis by health extension workers in Ethiopia: an ancillary cost-effectiveness analysis of community randomized trial. PLoS ONE. 2010;5(2):e9158. 10.1371/journal.pone.0009158 20174642 PMC2822844

[32] Nonvignon J, Chinbuah MA, Gyapong M, Abbey M, Awini E, Gyapong JO, et al. Is home management of fevers a cost-effective way of reducing under-five mortality in Africa? The case of a rural Ghanaian District. Trop Med Int Health. 2012 8;17(8):951–7. 10.1111/j.1365-3156.2012.03018.x 22643324

[33] Making choices in health: WHO guide to cost-effectiveness analysis. Geneva: World Health Organization; 2003.

[34] Greenspan JA, McMahon SA, Chebet JJ, Mpunga M, Urassa DP, Winch PJ. Sources of community health worker motivation: a qualitative study in Morogoro Region, Tanzania. Hum Resour Health. 2013;11(1):52. 10.1186/1478-4491-11-52 24112292 PMC3852396

[35] Kok MC, Dieleman M, Taegtmeyer M, Broerse JE, Kane SS, Ormel H, et al. Which intervention design factors influence performance of community health workers in low- and middle-income countries? A systematic review. Health Policy Plan. 2014 12 11; 10.1093/heapol/czu126 25500559 PMC4597042

[36] Takasugi T, Lee ACK. Why do community health workers volunteer? A qualitative study in Kenya. Public Health. 2012 10;126(10):839–45. 10.1016/j.puhe.2012.06.005 23036777

[37] Angwenyi V, Kamuya D, Mwachiro D, Marsh V, Njuguna P, Molyneux S. Working with Community Health Workers as ‘volunteers’ in a vaccine trial: practical and ethical experiences and implications. Developing World Bioeth. 2013 4;13(1):38–47. 10.1111/dewb.12015 23521823 PMC3662994

[38] McCollum R, Otiso L, Mireku M, Theobald S, de Koning K, Hussein S, et al. Exploring perceptions of community health policy in Kenya and identifying implications for policy change. Health Policy Plan. 2015 3 26;pii: czv007. 10.1093/heapol/czv007 25820367 PMC4724165

[39] Tulenko K, Møgedal S, Afzal MM, Frymus D, Oshin A, Pate M, et al. Community health workers for universal health-care coverage: from fragmentation to synergy. Bull World Health Organ. 2013 11 1;91(11):847–52. 10.2471/BLT.13.118745 24347709 PMC3853952

